# Polymorphism of apyrimidinic DNA structures in the nucleosome

**DOI:** 10.1038/srep41783

**Published:** 2017-01-31

**Authors:** Akihisa Osakabe, Yasuhiro Arimura, Syota Matsumoto, Naoki Horikoshi, Kaoru Sugasawa, Hitoshi Kurumizaka

**Affiliations:** 1 Laboratory of Structural Biology, Graduate School of Advanced Science and Engineering, Waseda University, 2-2 Wakamatsu-cho, Shinjuku-ku, Tokyo 162-8480, Japan; 2 Biosignal Research Center, Kobe University, 1-1 Rokkodai-cho, Nada-ku, Kobe, Hyogo 657-8501, Japan; 3 Research Institute for Science and Engineering, Waseda University, 2-2 Wakamatsu-cho, Shinjuku-ku, Tokyo 162-8480, Japan; 4 Institute for Medical-oriented Structural Biology, Waseda University, 2-2 Wakamatsu-cho, Shinjuku-ku, Tokyo 162-8480, Japan

## Abstract

Huge amounts (>10,000/day) of apurinic/apyrimidinic (AP) sites are produced in genomes, but their structures in chromatin remain undetermined. We determined the crystal structure of the nucleosome containing AP-site analogs at two symmetric sites, which revealed structural polymorphism: one forms an inchworm configuration without an empty space at the AP site, and the other forms a B-form-like structure with an empty space and the orphan base. This unexpected inchworm configuration of the AP site is important to understand the AP DNA repair mechanism, because it may not be recognized by the major AP-binding protein, APE1, during the base excision repair process.

In cells, more than 10,000 DNA apurinic/apyrimidinic (AP) sites are generated in genomic DNA each day, by spontaneous depurination/depyrimidination and the base-excision repair (BER) process^1^,^2^,^3^,^4^,^5^,^6^,^7^. Since unrepaired AP sites induce mutations and strand breaks in genomic DNA, leading to genomic instability^8^,^9^,^10^, the AP sites must be promptly removed and repaired. To this end, an AP endonuclease (APE) specifically incises the 5′ phosphodiester bond of the AP site^11^,^12^, and removes the AP site in coordination with the BER pathway^13^,^14^.

In eukaryotes, genomic DNA exists as chromatin, in which the histone octamer, composed of histones H2A, H2B, H3, and H4, wraps about 150 base pairs of DNA into a nucleosome^15^. The nucleosome structure generally inhibits interactions with DNA binding proteins, but human APE1 reportedly processes AP sites in nucleosomes at a reduced rate, as compared to a naked DNA substrate^16^,^17^. This indicates that the AP site is somehow recognized by APE1 without disrupting the nucleosome. However, the mechanism underlying this recognition has remained elusive, due to the lack of structural information about the AP site within a nucleosome.

To elucidate the structure of the nucleosomal AP site, we reconstituted nucleosomes containing an AP-site analog, tetrahydrofuran (THF). We then determined the crystal structure of a nucleosome containing THF at two symmetric sites at 2.5 Å resolution, and found that the DNA containing an AP site exhibits structural polymorphism, adopting both inchworm and B-form like configurations.

## Results

### Preparation of nucleosomes containing the AP-site analog, THF

**Figure 1:**
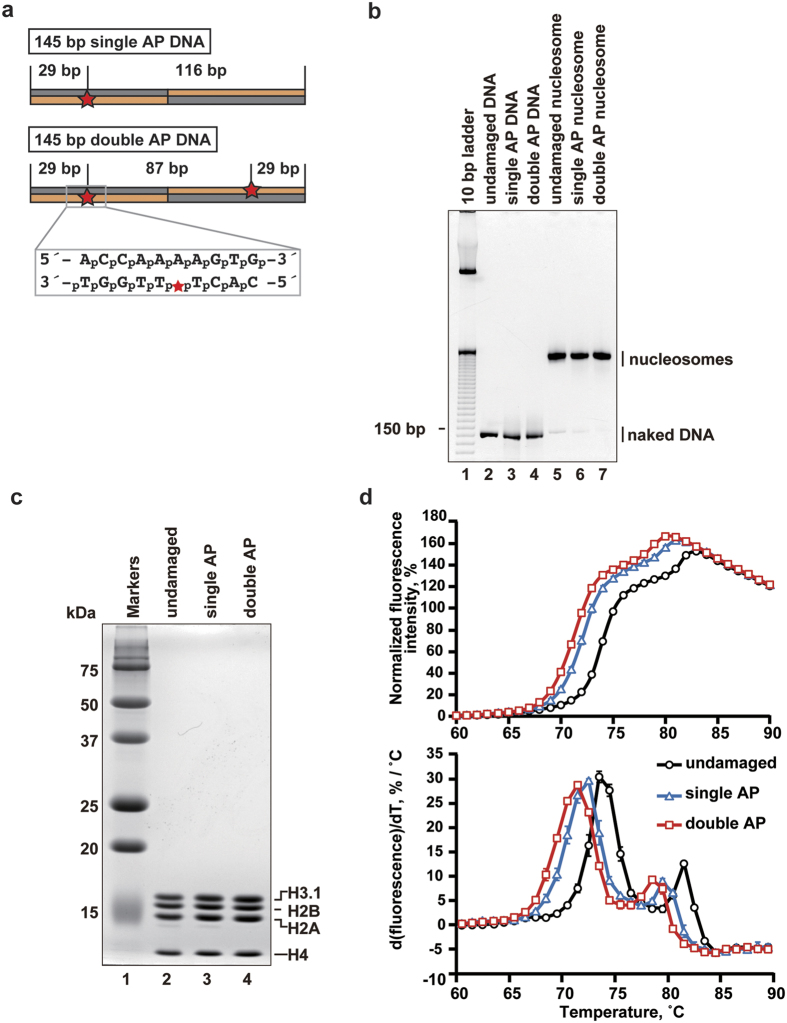
Preparation and thermal stability of nucleosomes containing an AP-site analog, THF. (**a**) Schematic representation of the DNAs containing THF at one or two symmetric sites. Red stars indicate the THF locations. The DNA sequence around the THF site is presented in a box. (**b**) Purified DNAs (lanes 2–4) and nucleosomes (lanes 5–7) were analyzed by non-denaturing 6% PAGE with ethidium bromide staining. Lane 1 indicates 10 base-pair DNA ladder markers. Lanes 2–4 indicate undamaged DNA, single AP DNA, and double AP DNA, respectively. Lanes 5–7 indicate purified nucleosomes containing undamaged DNA, single AP DNA, and double AP DNA, respectively. (**c**) The histone composition of the purified nucleosomes was analyzed by 18% SDS-PAGE with Coomassie Brilliant Blue staining. Lane 1 indicates molecular mass markers. Lanes 2–4 indicate nucleosomes containing undamaged DNA, single AP DNA, and double AP DNA, respectively. (**d**) Thermal stability curves of the undamaged, single AP, and double AP nucleosomes. Normalized fluorescence intensity was plotted at each temperature from 60 °C to 90 °C. Upper and lower panels indicate the thermal stability curves and the derivative values of the thermal stability curves of purified nucleosomes. Standard deviation values are shown (n = 3).

We prepared 145 base-pair DNA fragments containing THF, as an AP-site analog, at a single site or two symmetric sites (Fig. 1a). The AP site was introduced in the thymine residue located at position 29 from the 3′-end of the strand, in a 5′-T-(T)-T-T-3′ stretch. We then reconstituted the nucleosomes, containing a single AP site (single AP nucleosome) and two symmetric AP sites (double AP nucleosome), by the salt-dialysis method (Fig. 1b). In both the single AP and double AP nucleosomes, stoichiometric amounts of the core histones H2A, H2B, H3, and H4 were incorporated (Fig. 1c). We then tested the thermal stability of these AP nucleosomes in the presence of SYPRO Orange, which binds to thermally denatured histones^18^. In the experiment with a nucleosome containing undamaged DNA, the thermal dissociation of H2A-H2B and H3-H4 can be independently monitored as the first (Tm = 73–74 °C) and second peaks (Tm = 81–82 °C) under the experimental conditions used in this study (Fig. 1d, undamaged). Interestingly, in both the single AP and double AP nucleosomes, the H2A-H2B and H3-H4 dissociations occurred below the lower temperature, as compared to the undamaged nucleosome (Fig. 1d). The double AP nucleosome was less stable than the single AP nucleosome (Fig. 1d). These results indicated that the presence of the AP site reduces the nucleosome stability.

### Crystal structure of the nucleosome containing two symmetric AP sites

**Figure 2:**
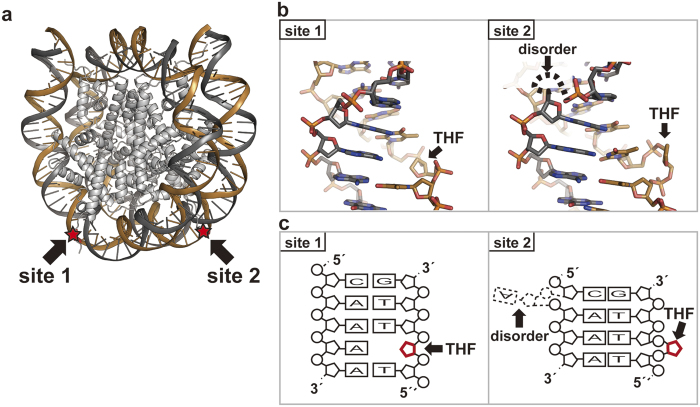
Crystal structure of the nucleosome containing two symmetric AP sites. (**a**) The double AP nucleosome structure. The two AP (THF) sites are represented by red stars, and AP site 1 and AP site 2 are indicated by arrows. (**b**) Close-up views of the DNA structures at AP site 1 and AP site 2. The location of the THF region is indicated by an arrow. (**c**) Schematic representation of the DNA structures at AP site 1 and AP site 2. The locations of the THF and the disordered regions are indicated by arrows.

**Figure 3:**
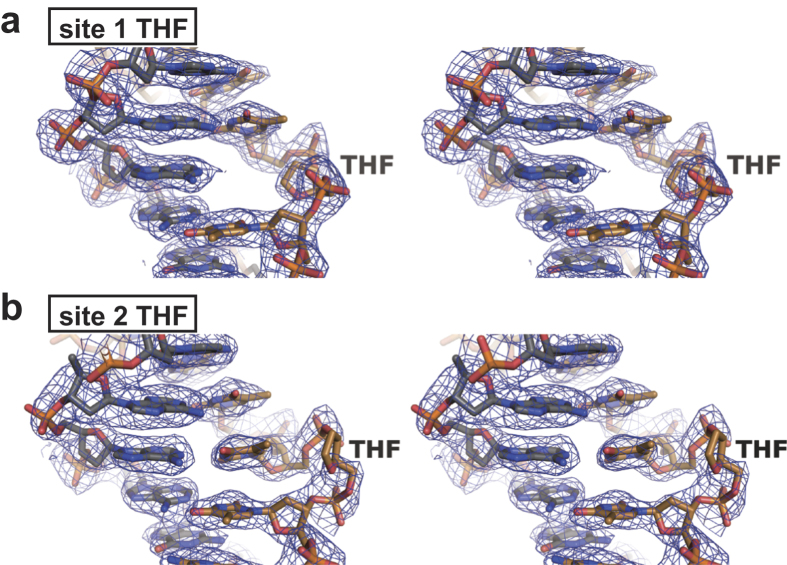
Stereoviews of the AP sites in the nucleosome. (**a**) The DNA structure at AP site 1. (**b**) The DNA structure at AP site 2. The 2mFo-DFc maps were calculated and contoured at the 1.0σ level.

To reveal the AP DNA structure in the nucleosome, we crystallized the double AP nucleosome (with a 145 base-pair DNA), and determined its crystal structure at 2.5 Å resolution (Fig. 2a and Supplementary Table 1). Surprisingly, we found that the DNA structures of the two AP sites (AP site 1 and AP site 2) are totally different. As expected, in AP site 1, the base pairing at the THF site had disappeared, and the free orphan base was accommodated within the double helix, as in B-form DNA (Figs 2a, 2b and 3a). In contrast, in AP site 2, the THF moiety was flipped out from the double helix, and the orphan base formed a base pair with the neighboring adenine base (Figs 2c and 3b). Importantly, the 5′-edge adenine base of the complementary A-A-A-A tract lost its base-pairing partner, and was disordered (Fig. 2c). As a result, the inchworm configuration, which contains two flipped out sites on each strand without an empty space and a free orphan base, is formed (Figs 2a, 2b and 3a). The inchworm configuration may form only in an A-A-A-A, T-T-T-T, C-C-C-C, or G-G-G-G tract, to establish new base pairing between the orphan and adjacent bases.

### DNA structures of the double AP nucleosome

**Figure 4:**
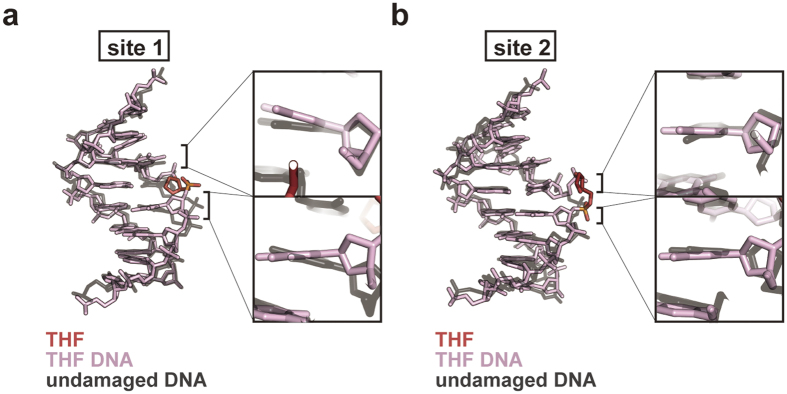
Comparison of the nucleosomal AP DNA structures with the undamaged nucleosomal DNA structure. Superimposition of the AP site 1 (**a**) and AP site 2 (**b**) structures of the double AP nucleosome with the corresponding regions of the undamaged nucleosome (PDB ID: 3AFA)^19^. The DNA strands containing the THF site are colored pink, and the THF moieties are colored red. Undamaged DNA strands are colored gray. Right panels show close-up views of the neighboring bases at the AP site 1 and the AP site 2 with the undamaged DNA in the nucleosomes.

We compared the DNA structures of AP site 1 and AP site 2 to that of undamaged nucleosomal DNA^19^. As shown in Fig. 4a, the DNA bases around the AP site 1 fit very well with the undamaged nucleosomal DNA structure, except for the region around the missing thymine base at the AP site. In this B-form like structure, the orphan adenine base is retained in the double helix by stacking interactions with neighboring bases (Fig. 3a). Although the base pairing had slipped by one base within the 5′-T-(T)-T-T-3′ stretch around the AP site 2 (inchworm), the structures of the base pairs are also similar to that of B-form DNA (Fig. 4b). This base-pairing slippage shortened the nucleosomal DNA from 145 to 144 base pairs. Therefore, the nucleosome is properly formed with a 144 base-pair DNA containing two protruding backbone sugar-phosphate sites (inchworm sites).

In the crystal with the nucleosome containing the 145 base-pair DNA, AP site 1 is located in close proximity to the neighboring nucleosome molecule, but AP site 2 is completely exposed to the solvent (Supplementary Fig. 1). These differences imply that the B-form like configuration may be induced by the crystal packing force. Our preliminary analysis also suggested that AP site 1 may adopt the inchworm configuration, rather than the B-form like configuration, in the nucleosome crystal with the 147 base-pair DNA (data not shown). In the nucleosome containing the 147 base-pair DNA, the DNA is more relaxed than that in the nucleosome containing the 145 base-pair DNA, which probably allows it to adopt the inchworm configuration, rather than the stretched B-form like configuration. These facts imply that the B-form like configuration may be induced by the stretching of the short 145 base-pair DNA or the crystal packing force. Therefore, the inchworm configuration may naturally occur in the nucleosome, if the AP site is formed in an A-A-A-A, T-T-T-T, C-C-C-C, or G-G-G-G tract in genomic DNA without DNA stretching and physical contacts. Further studies will be required to understand how the inchworm sites are formed, detected, and repaired by the BER pathway.

## Discussion

In the BER pathway, AP endonuclease (APE) specifically binds to the AP site, incises its 5′ phosphodiester bond^11^,^12^, and eventually removes the AP site^13^,^14^. When human APE1 binds to the AP site, the Arg177 residue intercalates into the major groove, and forms a hydrogen bond with the free orphan base^12^. The mutation of the Arg177 residue of APE1 drastically reduces its binding affinity to substrates and products^20^,^21^. These facts indicate that the free orphan base plays an important role to facilitate APE1 binding to the AP site in the DNA. However, in our crystal structure, the inchworm configuration found in AP site 2 lacked an available free orphan base for APE1 binding. This suggests that the AP site introduced in an A-A-A-A, T-T-T-T, C-C-C-C, or G-G-G-G tract may not be recognized by the BER machinery, and may cause a higher incidence of genomic instability.

In the present study, we found that the AP sites in the nucleosome are structurally polymorphic, with canonical and inchworm configurations in nucleotide-repeat regions. Notably, the inchworm configuration is preferentially formed in the nucleosome. In solution, both AP sites in the double AP nucleosome may adopt the inchworm configuration, and the B-form like configuration observed for AP site 1 may be induced by the crystal packing force or the stretched DNA conformation. Therefore, the inchworm configuration found in the present study may be the preferred structure for the AP site in chromatin, if it occurs in an A-A-A-A, T-T-T-T, C-C-C-C, or G-G-G-G tract.

The backbone and base conformations around the AP site 1 (with the B-form like configuration) are significantly distorted, as compared to those in undamaged nucleosomal DNA (Fig. 4a). Such DNA distortion is not obvious around the AP site 2 (with the inchworm configuration) (Fig. 4b). These facts suggest that the inchworm configuration may be more favorable in the nucleosome. The backbone and base distortions in the AP site 1 may reduce the stability of the local histone-DNA interactions around the AP site, and may promote the inchworm formation in the nucleosome.

We previously determined the structures of nucleosomes containing the ultraviolet light-induced cyclobutane pyrimidine dimer (CPD) and the pyrimidine-pyrimidone (6–4) photoproduct (6–4PP), which were inserted at symmetric locations similar to those in the double AP nucleosome^22^,^23^. The inchworm configuration of the damaged DNA site may be a specific characteristic of the AP lesion, because this structure was not observed in the CPD and 6–4PP nucleosomes^22^,^23^. This raises the question of how the AP site with the inchworm configuration is repaired in cells. A translesion DNA polymerase with high affinity for the looped out AP site may promote DNA synthesis and convert the inchworm configuration of the AP site, at least transiently, to the B-form like configuration in genomic DNA^24^,^25^. The mechanism by which the AP lesion with an inchworm configuration, which may not be properly recognized by the APE-BER pathway, is removed from genomic DNA in the absence of DNA replication presently remains enigmatic, and thus is an important puzzle to solve. Our preliminary analysis suggested that the B-form like configuration at the AP site may be induced by the stretched nucleosomal DNA and the crystal packing force. The inchworm configuration may preferentially form in the nucleosome, if the AP site of the nucleosomal DNA is relaxed and not physically contacted. Such nucleosomal DNA stretching may be induced by the actions of DNA-binding proteins and/or nucleosome remodelers involved in the inchworm AP repair in chromatin.

## Methods

### Preparation of DNA fragments containing THF

Three types of chemically-synthesized single-stranded 5′phosphorylated DNA 74-mers (oligo 1, 5′-GTTCA GCTGA ACATG CCTTT TGATG GAGCA GTTTC CAAAT ACACT (THF)TTGG TAGAA TCTGC AGGTG GATAT TGAT-3′; oligo 2, 5′-AACCA GCTGA ACATG CCTTT TGATG GAGCA GTTTC CAAAT ACACT TTTGG TAGAA TCTGC AGGTG GATAT TGAT-3′; oligo 3, 5′-AACCA GCTGA ACATG CCTTT TGATG GAGCA GTTTC CAAAT ACACT (THF)TTGG TAGAA TCTGC AGGTG GATAT TGAT-3′) and the complementary single-stranded DNA 71-mer (oligo 4, 5′-ATCAA TATCC ACCTG CAGAT TCTAC CAAAA GTGTA TTTGG AAACT GCTCC ATCAA AAGGC ATGTT CAGCT G-3′) were purchased from FASMAC, Japan. For the preparation of the DNA containing a single AP site, oligo 4 was annealed to oligo 1 and oligo 2. The two resulting double-stranded DNAs (dsDNA1 and dsDNA2) containing three base 5′-GTT and 5′-AAC overhangs, respectively, were ligated as described previously^22^. For the preparation of the DNA containing double AP sites, oligo 4 was annealed to oligo 1 or oligo 3, and the two resulting double-stranded DNAs (dsDNA1 and dsDNA3) were ligated.

### Purification of recombinant human histones

The DNA fragment encoding human histones H2A, H2B, H3.1, and H4 was inserted between the *Nde*I and *Bam*HI sites of the pET15b vector (Novagen). The N-terminally His_6_-tagged human histones H2A, H2B, and H3.1 were expressed in *Escherichia coli* BL21(DE3) cells, and the N-terminally His_6_-tagged human histone H4 was expressed in *E. coli* JM109(DE3) cells. These His_6_-tagged histones were purified by nickel-nitrilotriacetic acid (Ni-NTA) agarose column chromatography (Qiagen) under denaturing conditions, as described previously^26^. The His_6_-tag portion was removed by thrombin protease treatment, and histones without the His_6_ tag were further purified by Mono S column chromatography (GE Healthcare), as described previously^27^. The purified histones were freeze-dried and stored at 4 °C.

### Reconstitution of nucleosomes containing THF

The histone octamer was prepared according to the previously described method^27^. The nucleosomes were reconstituted with the 145 base-pair DNAs containing one or two THFs by the salt dialysis method, and were purified by non-denaturing polyacrylamide gel electrophoresis^19^.

### Thermal stability assay of nucleosomes

Thermal stability assays of the nucleosomes containing one or two THFs were performed by the previously described method^18^. The nucleosomes (final concentration 2.25 μM) were incubated with a temperature gradient from 26 °C to 95 °C, in steps of 1 °C/min, using a StepOnePlus^TM^ Real-Time PCR unit (Applied Biosystems), in 20 mM Tris–HCl (pH 7.5) buffer, containing 5x SYPRO Orange (Sigma-Aldrich) and 1 mM dithiothreitol. The raw fluorescence data were normalized by the previously described method^28^.

### Structure determination of the nucleosome containing THF

The purified nucleosome containing two THFs was crystallized by the hanging drop vapor diffusion method. The THF nucleosome solution (1 μL of 3.0 mg/mL) was mixed with 1 μL of 20 mM potassium cacodylate buffer (pH 6.0), containing 70 mM KCl and 105 mM MnCl_2_, and was equilibrated against 500 μL of reservoir solution, containing 20 mM potassium cacodylate (pH 6.0), 45 mM KCl, and 60 mM MnCl_2_, at 20 °C. The obtained crystals were soaked in a cryo-protectant solution, containing 20 mM potassium cacodylate (pH 6.0), 40.5 mM KCl, 54 mM MnCl_2_, 30% (+/−)-2-methyl–2,4-pentanediol, and 2% trehalose, and were flash-cooled with liquid nitrogen. X-ray diffraction experiments were performed at the SPring-8 (BL41XU) and Photon Factory (BL-1A and BL-17A) beamlines. Diffraction data for structure determination were eventually collected using the BL-17A station of the Photon Factory, at a 0.98000 Å wavelength. The diffraction data were scaled with the HKL2000 program, and were processed with the CCP4 program suite^29^. The structure of the THF nucleosome was determined by the molecular replacement method with the Phaser program^31^. The nucleosome structure (PDB ID: 3AFA) was used as the search model during the molecular replacement^19^. The structural refinement, model building, and Ramachandran statistics calculation were performed using the Phenix program suite, the COOT program, and the MolProbity program, respectively^32^,^33^,^34^. In the final structure, 98.54% of the amino acid residues are in the Ramachandran favored regions, and 1.46% of the amino acid residues are in the Ramachandran allowed regions. All structural graphics were drawn using the PyMOL program (http://pymol.org).

## Additional Information

**Accession code**: Coordinates and structure factors have been deposited in the Protein Data Bank, under the accession code 5JRG.

**How to cite this article**: Osakabe, A. *et al*. Polymorphism of apyrimidinic DNA structures in the nucleosome. *Sci. Rep.* **7**, 41783; doi: 10.1038/srep41783 (2017).

**Publisher's note:** Springer Nature remains neutral with regard to jurisdictional claims in published maps and institutional affiliations.

## Supplementary Material

Supplementary Information
