# Supplementary material for: Polymorphism of apyrimidinic DNA structures in the nucleosome
Source: Sci Rep. 2017 Jan 31;7:41783. doi: 10.1038/srep41783 (PMC5282573; doi:10.1038/srep41783)
Supplement: Supplementary Information [file srep41783-s1.pdf]

Supplementaly table and figure

Polymorphism of apyrimidinic DNA structures in the nucleosome

Akihisa Osakabe[1,5], Yasuhiro Arimura[1,5], Syota Matsumoto[2], Naoki Horikoshi[3], Kaoru Sugasawa[2], and Hitoshi Kurumizaka[1,3,4]

[1]Laboratory of Structural Biology, Graduate School of Advanced Science and Engineering, Waseda University, 2-2 Wakamatsu-cho, Shinjuku-ku, Tokyo 162-8480, Japan.

[2]Biosignal Research Center, Kobe University, 1-1 Rokkodai-cho, Nada-ku, Kobe, Hyogo 657-8501, Japan.

[3]Research Institute for Science and Engineering, Waseda University, 2-2 Wakamatsu-cho, Shinjuku-ku, Tokyo 162-8480, Japan.

[4]Institute for Medical-oriented Structural Biology, Waseda University, 2-2 Wakamatsu-cho, Shinjuku-ku, Tokyo 162-8480, Japan.

[5] These authors equally contributed to this work.

Supplementary table 1   Data collection and refinement statistics

	The nucleosome containing THF		
**Data collection**			
Space group	$P2_12_12_1$		
Cell dimensions			
a, b, c (Å)	99.491	108.773	169.571
α, β, γ (°)	90.000	90.000	90.000
Resolution (Å)	50-2.5 (2.59-2.50) *		
R_{sym} or R_{merge}	9.3 (41.8)		
$I / \sigma I$	17.5 (2.15)		
Completeness (%)	99.8 (99.8)		
Redundancy	9.2 (6.3)		
**Refinement**			
Resolution (Å)	49.2-2.50		
No. reflections	64243		
R_{work} / R_{free}	0.1929 / 0.2454		
No. atoms			
Protein	6119		
DNA	5900		
Ion	19		
Water	244		
B-factors			
Protein	31.6		
DNA	56.7		
Ion	57.5		
Water	35.6		
R.m.s. deviations			
Bond lengths (Å)	0.012		
Bond angles (°)	1.279		

*Values in parentheses are for highest-resolution shell.

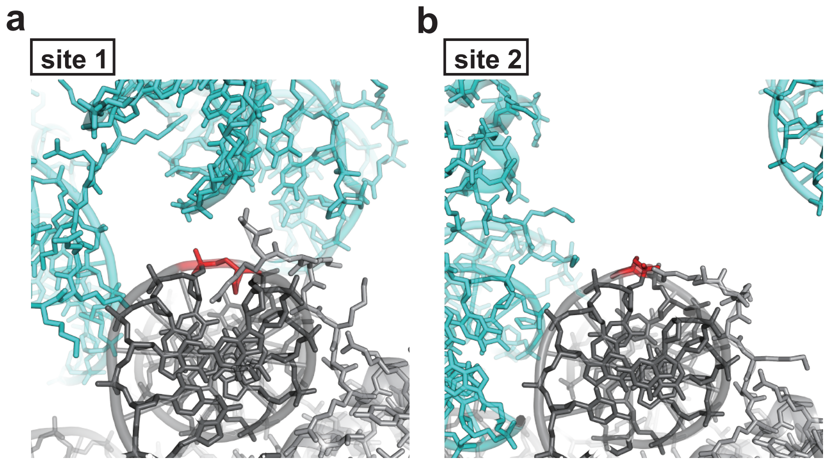

Supplementary Figure 1 Crystal packing interactions of the nucleosomal AP sites. **(a)** Close-up view of the AP site 1 (red), located in close proximity to the symmetry-related nucleosome molecule (light blue) in the crystal. **(b)** Close-up view of the AP site 2 (red). The symmetry-related nucleosome molecule is colored light blue.
